# Molecular natural history of breast cancer: Leveraging transcriptomics to predict breast cancer progression and aggressiveness

**DOI:** 10.1002/cam4.2996

**Published:** 2020-03-23

**Authors:** Daniel J. Cook, Jonatan Kallus, Rebecka Jörnsten, Jens Nielsen

**Affiliations:** ^1^ Department of Biology and Biological Engineering Chalmers University of Technology Gothenburg Sweden; ^2^ Wallenberg Center for Protein Research Chalmers University of Technology Gothenburg Sweden; ^3^ Department of Mathematical Sciences Chalmers University of Technology and University of Gothenburg Gothenburg Sweden; ^4^ Novo Nordisk Foundation Center for Biosustainability Technical University of Denmark Lyngby Denmark; ^5^ BioInnovation Institute Copenhagen N Denmark

**Keywords:** disease dynamics, patient heterogeneity, RNA‐seq, systems medicine

## Abstract

**Background:**

Characterizing breast cancer progression and aggressiveness relies on categorical descriptions of tumor stage and grade. Interpreting these categorical descriptions is challenging because stage convolutes the size and spread of the tumor and no consensus exists to define high/low grade tumors.

**Methods:**

We address this challenge of heterogeneity in patient‐specific cancer samples by adapting and applying several tools originally created for understanding heterogeneity and phenotype development in single cells (specifically, single‐cell topological data analysis and Wanderlust) to create a continuous metric describing breast cancer progression using bulk RNA‐seq samples from individual patient tumors. We also created a linear regression‐based method to predict tumor aggressiveness in vivo from bulk RNA‐seq data.

**Results:**

We found that breast cancer proceeds along three convergent phenotype trajectories: luminal, HER2‐enriched, and basal‐like. Furthermore, 31 296 genes (for luminal cancers), 17 827 genes (for HER2‐enriched), and 18 505 genes (for basal‐like) are dynamically differentially expressed during breast cancer progression. Across progression trajectories, our results show that expression of genes related to ADP‐ribosylation decreased as tumors progressed (while PARP1 and PARP2 increased or remained stable), suggesting the potential for a differential response to PARP inhibitors based on cancer progression. Additionally, we developed a 132‐gene expression regression equation to predict mitotic index and a 23‐gene expression regression equation to predict growth rate from a single breast cancer biopsy.

**Conclusion:**

Our results suggest that breast cancer dynamically changes during disease progression, and growth rate of the cancer cells is associated with distinct transcriptional profiles.

## Graphical abstract

Breast cancer is a continuous disease and RNA expression dynamically changes with disease progression. Additionally, cellular growth rate is encoded in RNA expression.

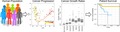

## 1 INTRODUCTION

Treatment for breast cancer results in an average 5‐year survival of 89.7%, stratified by stage at diagnosis ranging from 98.7% (for localized tumors) to 27.0% (for distant tumors).^1^ Doctors facilitating this fantastic treatment success in patients rely on multiple classification metrics to decide the specific treatment plan for each patient. These classification metrics include tumor staging, grading, and subtype identification.^2^ Breast cancer staging integrates information about tumor size and spread (local to the lymph nodes and distant to other organs) into one categorical score that is related to how long a patient has had a tumor, how fast the tumor is growing, and how easily the tumor metastasizes.^2^ Despite this convolution of growth time, growth rate, and metastatic potential and the relatively few staging categories, staging has clinical prognostic value.^3^ In contrast to the integrative staging score, breast cancer grading measures a single characteristic: proliferation index, commonly measured by mitotic index counting or Ki67 staining.^4^, ^5^, ^6^, ^7^ Like tumor staging, grading is assessed as a categorical classification––high grade or low grade—rather than a continuous score and also has clinical prognostic value.^6^ Unlike staging and grading, subtype classification involves grouping tumors based on molecular characteristics. Doctors subtype breast cancers for different treatments based on mutations^2^; estrogen receptor (ER), progesterone receptor, and human epidermal growth factor receptor 2 (HER2) protein expression status^6^; and more specific molecular subtyping, such as the PAM50 classification that groups tumors based on a panel of gene expression (ie, as luminal A, luminal B, HER2‐enriched, basal‐like, or normal‐like tumors).^8^ Such classification schemes bring with them a paradox: individual tumors have widely varying combinations of driver mutations and expression levels of subtype‐defining molecules, yet many tumors exhibit what have become known as the “hallmarks of cancer”, including evading the immune system, restructuring of the tissue microenvironment, increasing glycolysis, and shifting other characteristic metabolic processes.^9^ We hypothesize that such hallmarks represent a convergent phenotype for cancer cells where multiple mutation landscapes combined with a background molecular expression in pre‐cancerous cells lead to few transcriptional—and thereby functional—phenotypes.

Taking advantage of these increasingly stratified phenotypes, researchers continue to develop increasingly specific targeted therapies, including tamoxifen and aromatase inhibitors (for ER positive tumors)^10^ and Olaparib, a PARP inhibitor (for HER2‐negative tumors and tumors with germline BRCA mutations).^2^ Despite these and other advances, targeted therapies’ development and application do not consider tumor progression (as captured by tumor stage) or aggressiveness (as captured by tumor grade). Including these factors as considerations in targeted therapies is challenging because there is no clear understanding of how molecular regulation dynamically changes during tumor progression or across aggressiveness profiles. Further confounding this understanding of dynamic molecular regulation in breast cancer is that gene and protein expression levels across patients are highly variable due to differences in patient history, time of diagnosis, disease presentation, and DNA mutations.

In the past, these sources of variability have been treated largely as confounding variables obscuring biological function. In contrast to this view, leveraging the biological variability across individual cancer patients as a biological feature can result in a new understanding of disease progression and severity. In this study, we use gene expression across 1215 patients combined with analytical techniques adapted from tools originally developed to explore single‐cell biological heterogeneity and phenotype development to predict a molecular natural history of breast cancer progression in different subtypes of breast cancer from samples taken from individual patients. We also develop a transcriptomic signature of cellular growth rate, use it to predict tumor doubling time in individual patients, and investigate the clinical applicability of growth rate estimation in breast cancer patients.

## 2 MATERIALS AND METHODS

### 2.1 RNA‐seq data acquisition and normalization

Two datasets were primarily used in this study: the The Cancer Genome Atlas (TCGA) breast cancer (BRCA) study^11^ and The Sweden Cancerome Analysis Network for breast cancer (SCAN‐B) study.^12^ For the TCGA BRCA study, tumor resection samples were collected from treatment‐naïve patients and frozen “soon after surgery” for later sequencing, as described previously,^11^ see also (https://cancergenome.nih.gov/cancersselected/biospeccriteria). For the SCAN‐B study, treatment‐naïve patients and patients receiving neoadjuvant therapy were enrolled prior to surgery.^12^ At the time of presurgical biopsy, additional study biopsies were taken, placed in RNA‐later (Ambion) and frozen for later sequencing, as described previously.^12^

Primary BRCA and paired‐normal breast RNA‐seq data were downloaded from the TCGA BRCA project (available online at https://portal.gdc.cancer.gov/projects/TCGA-BRCA). For data projection and differential expression analyses, HT‐seq counts were normalized using the size factor normalization technique available in DESeq2.^13^ For development of growth rate and mitotic index regression equations, raw FPKM values were normalized by conversion to TPM by dividing each sample by the sum of its FPKM values.^14^

### 2.2 Single‐cell topological data analysis

Single‐cell topological data analysis (sc‐TDA) was performed on the log‐transformed RNA‐seq data from TCGA BRCA whole‐tissue, single patient samples using the python implementation available on Github (https://github.com/CamaraLab/scTDA).^15^ Only the top 250 highest weighted genes from a PCA analysis of these log‐transformed data were used to determine the scTDA projection. Following scTDA projection and classification, we excluded normal‐like tumors from our analysis because of low numbers of patients with this phenotype.

### 2.3 Consensus Wanderlust to predict a CPS

**FIGURE 1:**
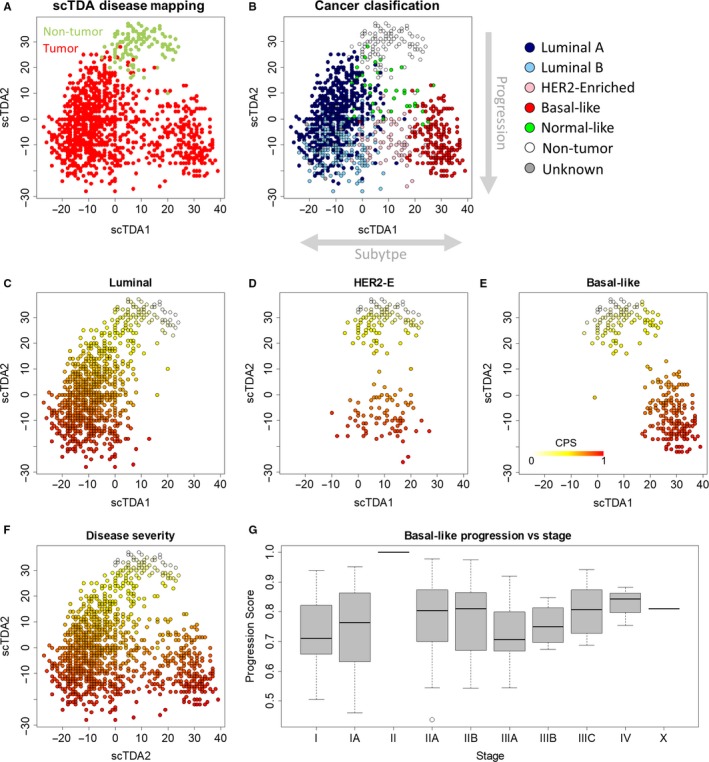
Mapping BRCA treatment‐free progression. A, Double PCA/scTDA projection of breast cancer and pair‐matched nontumor breast RNA‐seq data showed gene expression separated nontumor from tumor samples. Tumor samples further separated into three groups. Each sample was represented by one point. B, ScTDA axis 1 separated tumors based on PAM50 classification. ScTDA axis 2 appeared to separate tumors based on similarity to nontumor tissue, suggesting that scTDA2 may capture cancer progression. C‐E, CPS for each progression trajectory estimated using consensus Wanderlust. F, Composite CPS for all progression trajectories. G, Relationship between cancer stage and CPS for basal‐like breast cancer. CPS tended to increase with increasing cancer stage. CPS, Cancer Progression Score; PCA/scTDA, principal component analysis/single‐cell topological density analysis

We developed a consensus Wanderlust algorithm based on the Wanderlust algorithm designed to predict time‐series behavior from “snap‐shot” data describing single cells.^16^ As input data to Wanderlust, we used the coordinates from the PCA‐scTDA‐projected BRCA patients (see Figure 1), for each predefined progression trajectory. This combination of data projection and the Wanderlust algorithm uses a graph‐based approach to predict a progression trajectory through states (or samples) based on a random walk through similar states from a designated starting point. For our consensus Wanderlust, we iteratively ran the Wanderlust algorithm using each nontumor sample as a starting point. This resulted in *m* Wanderlust scores for each data point, where *m* is the number of nontumor samples. We then took the median Wanderlust score as the Cancer Progression Score (CPS) for each sample. Thus, our CPS is computed based on the assumption that tumors begin with expression profiles similar to those of stromal tissue and deviate from that expression profile as a tumor persists in the stroma. For our analysis, we used the R implementation of Wanderlust available in the “uSort” package.^17^

### 2.4 Differential gene expression analysis

Following development of the CPS, samples were grouped into discrete CPS levels of size 0.1 from 0 to 1. In all progression trajectories, this resulted in imbalanced groups, so the 0.0‐0.1 group was combined with the 0.1‐0.2 group and the 0.9‐1.0 group was combined with the 0.8‐0.9 group. Differential expression between stroma and cancer was calculated using the Wald test feature in DESeq2 for each cancer PAM50 subtype (Luminal A, luminal B, HER2‐enriched, and basal‐like) with all nontumor tissues acting as the control for each subtype (ie, using nonpaired statistics). Dynamic differential expression during cancer progression was calculated using a likelihood ratio test comparing the likelihood that gene expression is dependent on CPS to the likelihood that gene expression is independent of CPS using the likelihood‐ratio test (LRT) feature in DESeq2 for each progression trajectory (luminal, HER2‐enriched, and basal‐like). False detection rate was estimated using the Benjamini‐Hochberg procedure, and genes were denoted as differentially expressed if the FDR corrected *P*‐value was below .05. This level was chosen to limit false positives while also identifying a large number of differentially expressed genes to be useful for downstream analysis. No fold‐change threshold was used to identify differentially expressed genes.

### 2.5 GO overrepresentation test

We used the online tool PANTHER version 13.1 (http://www.pantherdb.org/tools/index.jsp) to identify GO Biological Processes overrepresented in gene co‐expression modules.^18^ We performed a statistical overrepresentation test using default parameters and metabolic genes measured in the TCGA BRCA data and included in the generic human metabolic model HMR2.0^19^ as a reference background and “GO biological process complete” as the annotation dataset or using all human genes as the reference background and “GO biological process complete” as the annotation dataset. GO‐terms were considered overrepresented only if FDR‐corrected *P*‐values were below <.05. Only overrepresented, not underrepresented, GO terms were considered in our analysis.

### 2.6 Development of the growth rate and mitotic index regression equations

We first obtained previously published RNA‐seq data and specific doubling times from 38 cell lines included in the NCI60 cell line panel (available online at https://data.broadinstitute.org/ccle/).^20^, ^21^ We next converted the RNA‐seq data to TPM (from FPKM) by dividing reads from each sample by the total number of reads in that sample and multiplying by 10^6^. We removed genes from the analysis that were not expressed in at least 38 out of 42 total samples. We then found genes whose log‐transformed expression levels correlated with log‐transformed growth rate (absolute value of Pearson correlation ≥0.64), resulting in 23 genes highly correlated with growth rate. We used standard linear regression to develop an equation relating the log‐transformed expression level of these 23 genes with log‐transformed growth rate (see Table S3). This method is based on the approach taken by Diener et al.^21^

We predicted fraction of cells actively proliferating (ie, mitotic index) by calculating the PCNA meta‐gene score in each of the 405 breast cancer samples available in the SCANB cohort of patients (GEO accession: http://www.ncbi.nlm.nih.gov/geo/query/acc.cgi?acc=GSE96058) that had associated Ki67 quantification.^22^, ^23^, ^24^ Linear regression was then used to associate Ki67‐based mitotic index with log‐transformed PCNA metagene expression (see Tables S4 and S5). The predicted growth rates were then corrected for fraction of cells actively proliferating as shown below.

(1) $$\mu_{\text{corrected}}=\mu_{\text{uncorrected}}\times \text{proliferation fraction}$$

(2) $$\text{Cellular doubling time}=\frac{\ln\left(2\right)}{\mu_{\text{corrected}}}$$

(3) $$\text{Tumor doubling time}=\frac{\text{cellular doubling time}}{\text{proliferation fraction}}$$

where *μ* is the specific cellular growth rate (measured in 1/h), the cellular doubling time is equivalent to the cellular maximum proliferative doubling time, and the tumor doubling time is the time required for the tumor burden to double.^25^

### 2.7 Statistical analysis of growth rate and mitotic index predictions

We calculated an *r* ^2^ value adjusted for number of parameters included in the RNA‐growth rate regression model to estimate how well our growth rate equation matched measured growth rates for our training data using the *lm* function in R.^26^ For our test data, we calculated how well our RNA‐estimated growth rate matched experimental values for our test data using Pearson correlation between calculated and measured values. We again used the *lm* function to estimate the correlation between the PCNA metagene expression and Ki67 values for the Ki67 index predicting equation (again adjusted for number of model parameters).

### 2.8 Survival analysis

Cancer predicted growth rate and CPS was subjected to standard Cox proportional hazards regression model and Kaplan‐Meier survival analyses using the “survival” package in R with default options, which uses a log‐rank test to calculate a *P*‐value comparing survival curves in Kaplan‐Meier survival analyses.^26^, ^27^, ^28^

### 2.9 SCAN‐B data processing

An additional ~3700 patient samples were included from the SCANB dataset.^12^ Because this dataset is missing many genes included in the BRCA TCGA data, we predicted growth rate and CPS in the SCANB samples based on gene expression correlation with TCGA BRCA data. Briefly, growth rate was predicted by correlating growth‐associated genes (absolute Pearson correlation value ≥0.6 between gene expression and TCGA growth rate) between each SCANB sample and all TCGA BRCA samples. The growth rate of the TCGA sample with the highest correlation was assigned to that SCANB sample. A similar procedure was followed for predicting the SCANB CPSs, using CPS‐dependent differentially expressed genes within each PAM50 subtype with the following exception. For HER2‐enriched cancers, the small samples size in TCGA BRCA led to the detection of few genes differentially expressed with cancer progression. Therefore, we intersected the differentially expressed genes in common from both luminal and basal‐like cancer, resulting in 16 552 genes differentially expressed in both tumor types. We included these genes in the correlation analysis for HER2‐e tumors from the SCANB dataset. Not all of the genes identified from the TCGA dataset‐matched genes measured in the SCANB dataset (resulting in a 14 254 gene overlapped for luminal tumors, a 8440 gene overlap for HER2‐e tumors, and a 8361 gene overlap for basal‐like tumors used for the correlation‐based analysis).

### 2.10 Software availability

The computer code necessary to reproduce the results of this study are available as supplemental files. Additionally, an R implementation of the regression equations for estimating growth rate (described above) and all code necessary to reproduce the results of this study, including text files containing the genes used in the regression equations and their coefficients, are available on Github (https://github.com/SysBioChalmers/Cancer-progression).

### 2.11 Data and materials availability

Data used in this study are available from TCGA (project: BRCA) and on the Gene Expression Omnibus website (GEO accession: http://www.ncbi.nlm.nih.gov/geo/query/acc.cgi?acc=GSE96058).

## 3 RESULTS

### 3.1 Developing a pseudo‐time series of RNA expression during breast cancer progression

Because of our hypothesis of a convergent transcriptional phenotype for cancer cells despite multiple mutation profiles, we used transcriptional data without any associated mutation data to explore breast cancer progression and aggressiveness. For the purpose of this study, we define progression as how a single tumor progresses from a small number of neoplastic cells in the primary tumor site to an increasing number of neoplastic cells in the primary tumor site that can begin to spread throughout the body. Our definition of progression refers only to natural progression in the absence of clinical intervention does not relate directly to recurrence, effectiveness of treatments, or clinical outcome. We downloaded and normalized RNA‐seq data for 1215 breast cancers and matched nontumorous breast tissue from the breast cancer (BRCA) project in The Cancer Genome Atlas (TCGA).^11^ We used a principal component analysis and single‐cell topological density analysis (PCA/scTDA) double projection technique to reduce the effects of noninformative gene expression in the data on subsequent analyses and to project the RNA‐seq data for each patient into a lower‐dimensional Euclidean space, where each point represents the transcriptome of a single sample and the distance between two points is related to the similarity of their transcriptomes (Figure 1A).^15^ This double projection technique showed separation between the coordinated gene expression of BRCA tumor and nontumorous breast samples; the organization of transcriptional phenotypes within BRCA tumor samples, however, remained unclear. We, therefore, annotated patient samples based on their PAM50 subtype classification (Figure 1B).^8^, ^29^ This classification scheme showed that the first scTDA axis separated patient tumors based on PAM50 subtype. This is not surprising, as gene expression was used as the basis for developing the PAM50 classification scheme. Based on the separation in scTDA‐space, we used a three‐subtype classification in our subsequent analyses: luminal, HER2‐enriched, and basal‐like.

The second scTDA axis appeared to separate patients based on their dissimilarity from nontumorous tissue. In addition, this dissimilarity is unidirectional: nontumor samples appear at the top of the plot, while tumor samples are projected predominantly below healthy samples, as opposed to other possible organizations like radiating outwards. We propose that this axis captures broadly the coordinated gene expression changes that occur as a cancer progresses from early incidence of tumor formation to a more advanced tumor, which is supported by the unidirectional projection indicating that one factor is driving this differentiation from healthy tissue in all tumor samples. The assumption underlying this proposal is that tumors begin with gene expression profiles similar to stromal tissue and continue to differentiate their gene expression in a consistent way across tumor subtypes as they persist in the stoma. To formalize this proposal and more precisely characterize breast cancer progression, we developed a modified version of the Wanderlust algorithm to construct a pseudo‐time series of primary tumor progression based on RNA‐seq data from clinical patients. Briefly, the Wanderlust algorithm is a graph‐based approach to ordering samples along a progression or developmental trajectory based on a random walk along an ensemble of nearest‐neighbor graphs, originally developed for single cells.^16^ Our modification applies Wanderlust multiple times using each nontumor sample as a starting point for the algorithm and averages each point's Wanderlust score across all applications to calculate a CPS. This CPS represents the progression of the primary tumor in the absence of treatment. In other words, we use the treatment‐naïve samples collected at different points in primary breast cancer's natural history (when the cancer was caught in individual patients) to construct a “pseudo‐natural history” of breast cancer, which is represented by the CPS. We applied our consensus Wanderlust on each of the three progression subtypes (or trajectories) individually (Figure 1C‐E) and developed a data‐driven, complete disease progression landscape for primary breast cancer (Figure 1F). We then tested how our CPS related to discrete cancer staging and found that CPS tended to increase with increasing cancer stage (Figure 1G and Figure S1). There appeared to be a nonmonotonic relationship between CPS and cancer stage: CPS generally seemed to increase with increasing stage within stage I and II cancers and within stage III and IV cancers (Figure S1); however, CPS seemed to decrease when progressing from stage II to stage III tumors. This variability could be caused in part by the multiple factors contributing to cancer staging: both tumor size and spread, whereas the CPS only captures the residence time of a tumor within a stromal environment. Whether this is the only factor contributing to the nonmonotonic relationship or not, we emphasize that our CPS is a data‐driven starting point to predict dynamic changes that could be occurring during breast cancer progression. Further longitudinal studies are required to explore any potential dynamic changes occurring in breast cancer that we predict as a result of our CPS.

### 3.2 Predicting differential gene expression during cancer progression

Having developed a CPS, we next investigated how gene expression changed over the predicted course of breast cancer progression. Differential gene expression between tumor and stroma has been comprehensively studied elsewhere, including analyses using the same dataset as this work.^11^ Our analysis largely captured the same features of breast cancer previously described, including increased expression of genes related to DNA replication, WNT signaling, and response to unfolded protein and decreased expression of genes related to fatty acid oxidation, cytokine and chemokine production, response to ethanol, and—in luminal B tumors––response to ketones (Figure S2).

After the previous standard differential expression analysis between tumor and stroma, we next focused on investigating how gene expression dynamically changes during breast cancer progression. Lumping patients with similar CPS values into “progression time steps” allowed us to use standard dynamic differential expression techniques to find genes whose expression changed during cancer progression; specifically, we used the LRT in DESeq2.^13^ We found that a large number of genes showed dynamic differential expression during cancer progression: 31 296 genes for Luminal tumors, 17 827 for HER2‐enriched tumors, and 18 505 genes for Basal‐like tumors (see Data S1).

**FIGURE 2:**
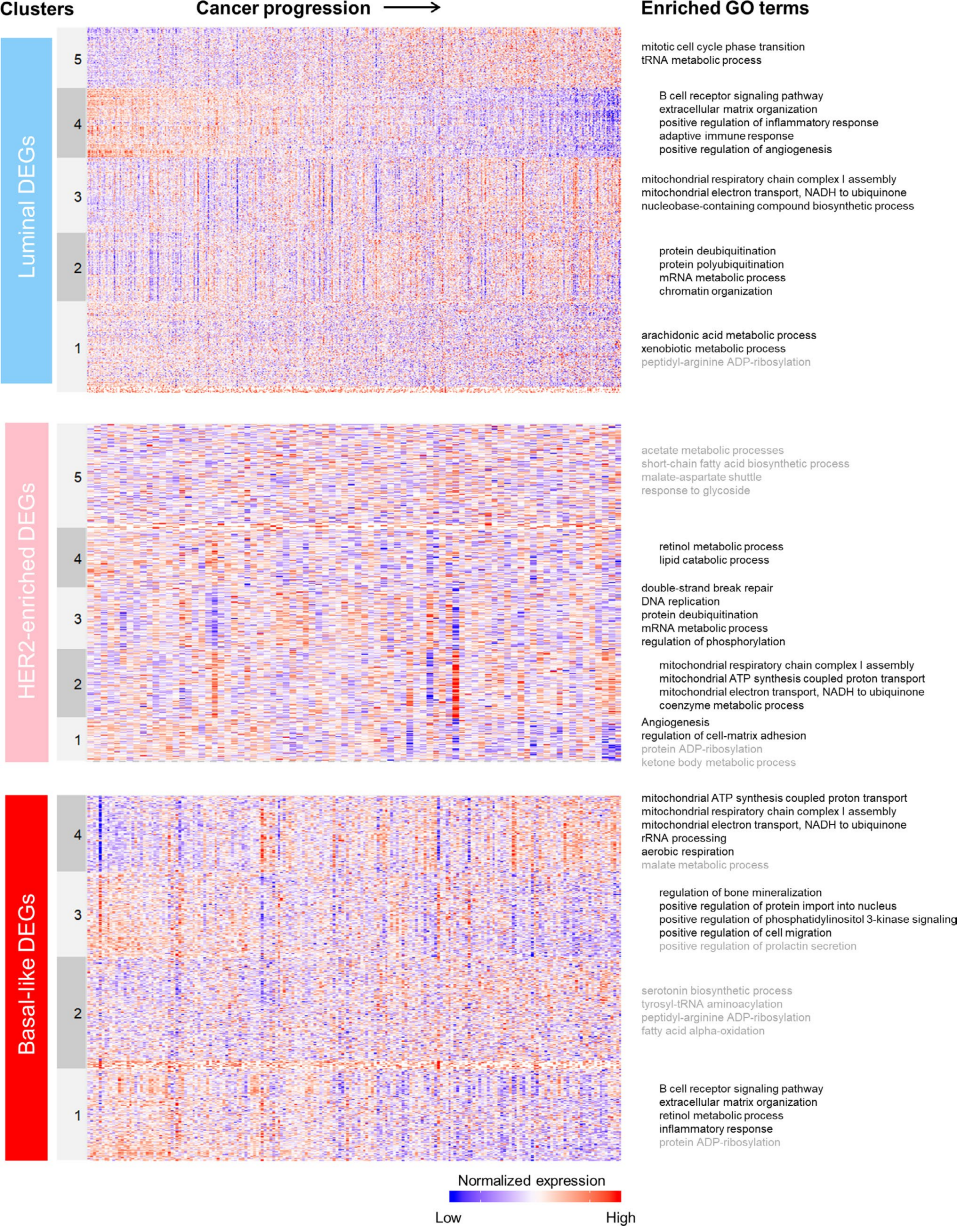
GO Term overrepresentation analysis of dynamically differential gene expression profiles of breast cancer for each progression trajectory using PANTHER.^18^ Overrepresented GO terms were found using metabolic genes measured in the TCGA BRCA data and included in the generic human metabolic model HMR2.0^19^ as a reference background and “GO biological process complete” as the annotation dataset. Terms written in grey were found using all human genes as the reference background and “GO biological process complete” as the annotation dataset

Given the large number of genes dynamically differentially expressed, we were especially interested in investigating how gene expression of genes related to metabolic function dynamically changed in tumors during predicted breast cancer progression, an approach made possible by our calculated CPS. We found that differential gene expression of metabolism‐related genes during cancer progression was dominated by relatively few co‐expression modules (Figure 2). The computer code required to reproduce our results is available on Github (https://github.com/SysBioChalmers/Cancer-progression) and as a supplement to this study (using the Supplemental file Predict‐Progression‐BRCA.R, which calls: Predict‐Growth‐Rates.R, Predict‐Mitotic‐Index.R, ki67_pred_lm.txt, nci60_growthRegressionCoeff_updated.txt, and venet_2011_PcnaGenes.txt). Additionally, an online tool allowing for more detailed exploration of these data is available at https://cookd.shinyapps.io/Cancer_progression/. More differentially expressed genes and more nuanced expression patterns were found in the luminal trajectory because this trajectory had more patient data and a more balanced distribution of CPS, leading to a more powerful statistical analysis. As breast cancer progresses, all subtypes showed increasing expression of genes related to cell cycle, DNA replication and mitochondrial function; they also showed decreasing expression of genes related to angiogenesis, extracellular matrix organization, and inflammation (Figure 2). In addition, all subtypes showed decreased expression of genes related to ADP ribosylation (Figure 2), which may influence the efficacy of PARP inhibitors among patients with more progressed tumors; although expression of the primary targets of PARP inhibitors (PARP1 and PARP2) either remained level or increased in expression during cancer progression. In HER2‐enriched and basal‐like tumors, progression was associated with a decreased expression of genes related to retinol metabolic processes. This analysis highlights cancer as a dynamic disease that restructures its microenvironment and dynamically responds to changes in the underlying tissue and immune system. Furthermore, our results support the idea that, early in cancer progression, cancers express higher levels of transcripts related to angiogenesis and restructuring the tissue microenvironment than at later stages, suggesting that anti‐angiogenesis‐based treatments will likely be more effective during the early stages of breast cancer development than at later stages.

### 3.3 Predicting cancer growth rates across multiple cancer types using an RNA‐based signature

**FIGURE 3:**
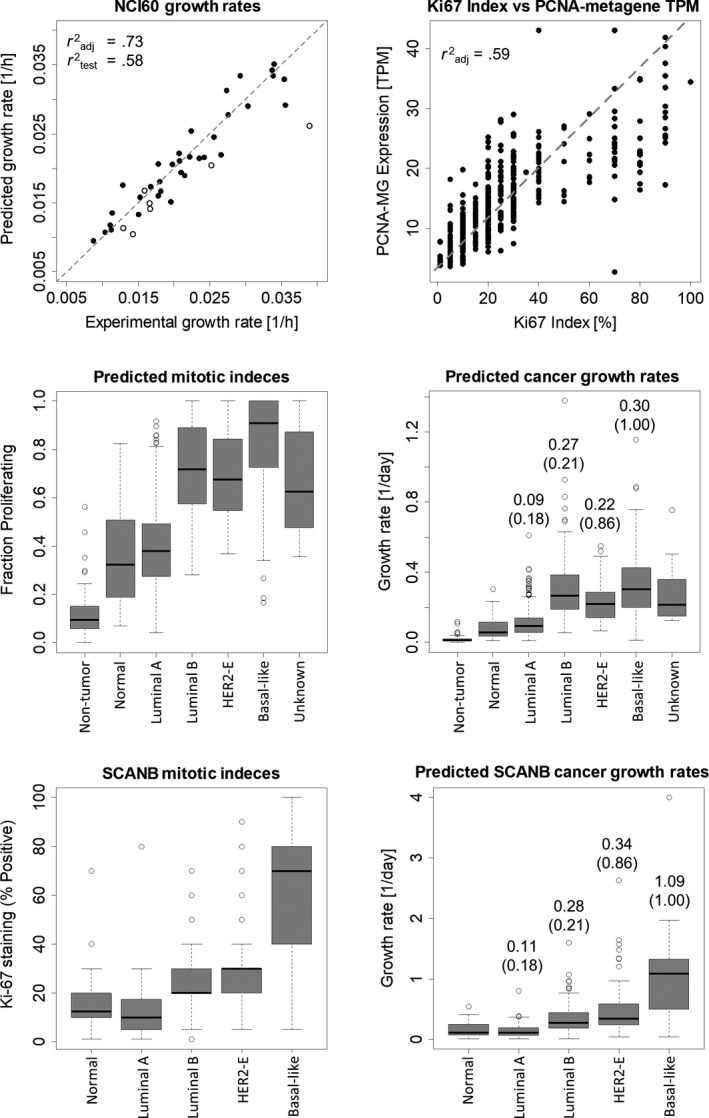
Predicting growth rates from RNA‐seq data. A, We used data from the NCI60 cell line panel to develop a linear regression equation to predict tumor growth rates from RNA‐seq data. B, We next used data from the SCANB breast cancer transcriptomic study to relate PCNA metagene expression to mitotic index (or Ki76 staining). These regression equations allowed us to predict (C) mitotic index and (D) specific growth rates from TCGA BRCA dataset and (E) mitotic index and (F) specific growth rates from the SCANB breast cancer dataset

Next, we attempted to use gene expression to predict tumor growth rates (and thereby doubling times). We used in vitro RNA‐seq data from the NCI60 panel of cell lines to develop a regression equation predicting cell growth rate from the expression levels of 23 genes, using 42 cell lines from the NCI‐60 panel with high‐quality transcriptomics matched to growth rates in the same cultures.^20^, ^21^ We randomly selected 38 out of 42 of the cell lines as training cells and were able to predict growth rate accurately in the remaining test cells (Figure 3A). When we tested the growth rate equation in the TCGA BRCA data; however, the absolute growth rates predicted were much higher than observed pathologically. For example, the median predicted tumor volume doubling time was approximately 2.4 days across TCGA BRCA dataset, which is much faster than even the fastest observations of tumor volume doubling time (median of 14.5 days for breast cancer).^30^

This led us to a long‐standing question in the field of tumor biology: what can explain the discrepancy between high in vitro growth rates and slow in vivo tumor doubling times.^21^, ^31^ Recently, researchers addressed this discrepancy in human patients using BrdU incorporation to calculate maximum proliferation doubling time of prostate cancer cells and comparing this maximum cellular doubling time to the tumor doubling time, measured by prostate‐specific antigen levels in the blood.^25^ Their data show that maximum cellular doubling time is an order of magnitude faster than tumor doubling time. The authors speculate that a high rate of tumor cell death may contribute to this timing mismatch. In contrast to this explanation, we propose that this timing mismatch is caused by only a fraction of tumor cells replicating at any given time, a well‐known feature of cancer. In this case, the maximum cellular doubling time is only achieved by a fraction of tumor cells. We found that incorporating the fraction of proliferating cells (as measured by BrdU incorporation) with the maximum cellular doubling time led to a prediction of tumor doubling time that matched experimentally observed doubling times closely in this prostate cancer dataset (Table S1).

Our regression equation calculates the growth rate of cells assuming that all cells from the sequenced population contribute equally to gene expression levels. Furthermore, because the regression was derived from cells in vitro, it assumes that all cells are growing at equal rates. We therefore introduced an additional parameter in our regression model corresponding to the fraction of cells in a tumor actively proliferating at any given time (in practice measured by Ki67 staining, BrdU incorporation, or mitotic index counting). The BRCA TCGA data, however, does not include a measure of fractional proliferation. We therefore derived a gene expression‐based equation that uses a previously identified “PCNA metagene” to predict fractional proliferation, using the SCANB breast cancer transcriptomic study for calibration (Figure 3B).^12^, ^22^, ^23^, ^24^

We then tested how well our gene expression‐based regression equations predicted mitotic index and experimental growth rates across breast cancer subtypes in the TCGA BRCA dataset (Figure 3C,D). We predicted mitotic indexes and growth rates that increase with increasingly severe clinical subtypes (normal‐like, luminal A, luminal B, HER2‐enriched, then basal‐like) compared to experimentally measured specific growth rates.^32^ These results were not specific to the TCGA BRCA dataset; the SCANB dataset showed the same trend, with an even greater agreement between predicted and experimental specific growth rates across tumor subtypes (Figure 3E,F). We also tested our regression equations across seven cancer types from TCGA, with some agreement between predicted and experimentally observed tumor volume doubling time for six cancer types (Table S2).

### 3.4 Combining predicted disease severity and growth rate to predict survival

We investigated if CPS or our growth rate prediction could predict overall patient survival in TCGA’s BRCA dataset. Specifically, we tested the association of survival with CPS and predicted growth using a Cox proportional hazards regression model. We found that the null hypothesis of lack of association could not be rejected at the 0.05 level for either predictor, and thus we found no evidence for association in this data set (*P* = .059 for CPS and *P* = .84 predicted growth). We then tested a larger dataset consisting of ~3700 breast tumor samples. We found a possible association between predicted growth rate and survival (*P* = .011) and no evidence for association between CPS and survival (*P* = .11) when considering all PAM50 subtypes together. When considering each progression trajectory individually (ie, Luminal, HER2, or Basal), this association was not significant at the 0.05 level.

Because chemotherapy is thought to be most effective for fast‐growing tumors and because it is often a first‐line treatment for Basal‐like breast cancer, we tested whether predicted growth rate was associated with survival in chemotherapy‐treated patients with Basal‐like breast cancer. A univariate analysis indicated that growth rate could be associated with survival when stratifying by median growth rate (Figure S3). A multivariate analysis using a Cox proportional hazards regression model, however, failed to confirm this association at a 0.05 confidence level.

## 4 DISCUSSION

This work takes steps to investigates an often‐overlooked aspect of breast cancer biology: primary breast cancer is a dynamic disease. Breast cancer dynamics are widely recognized when related to vascularization (early on in tumor development), tumor growth (during tumor development), and eventually metastasis (late in breast cancer's natural history). Expanding this view of dynamic events related to tumor progression, our work showed that gene expression in primary breast cancer appears to change dynamically throughout the natural history of breast cancer. We found an unexpectedly large number of genes differentially expressed during breast cancer progression across subtypes: in luminal tumors, 55% of measured transcripts were dynamically differentially expressed during progression (31 296 out of 56 830 transcripts measured by TCGA consortium); in HER2‐enriched tumors, 31% were dynamically differentially expressed (17 827 transcripts); and in basal‐like tumors, 32% were dynamically differentially expressed (18 505 transcripts). Further research is required to understand which of these changes are caused by disease progression, which may drive progression, which are not relevant to tumor progression, and how these changes at the transcriptional level are encoded in the proteome and functionally relevant. If such large‐scale dynamic transcriptomic changes are confirmed in further experiments, it would require a rethinking of how we view breast cancer to understand it as a dynamic system constantly responding to and changing its environment. Such a reconsideration of a tumor as a “static” disease is already occurring through thinking about how breast tumors relate to the immune system, to vascularization, and to the tumor microenvironment.^33^, ^34^, ^35^ Specifically, we believe that longitudinal, RNA‐seq studies of mice with xenograft tumors in individual mice is a natural next step to further investigate the findings presented in this work.

During our dynamic differential expression analysis, we were particularly interested to find that our inferred tumor progression RNA‐seq profiles indicate that protein ADP‐ribosylation activity may be decreasing as tumors progress in severity. Reduced ADP‐ribosylation activity could be because advanced tumors have less of a need for ADP ribosylation. Based on this finding, we predict that the newly approved PARP inhibitors for breast cancer treatment will have a greater effect on early‐stage breast cancer tumors than on late‐stage tumors.^2^ Although, we note that PARP1 and PARP2 (the primary targets of PARP inhibitors) increased or remained stable throughout tumor progression, which may lead to a stable effect of PARP inhibitors across tumor stages. We therefore recond that further studies evaluate PARP inhibitor effectiveness across tumor stages.

In addition to investigating tumor progression using a continuous metric, we also predicted tumor growth rates from RNA‐seq data. Previous studies estimating cancer cell growth and tumor volume doubling time have attributed the mismatch between maximal cancer growth rate, like that seen in vitro, and tumor volume doubling time, as seen in vivo, to lack of nutrient availability or increased cell death.^25^ Our work, however, points to the fraction of cancer cells proliferating in a tumor at a given time (or the mitotic index) as the important factor governing the difference between cellular doubling times and tumor doubling times. Our growth rate predictor combines an RNA‐based regression equation predicting growth rate in vitro with an estimator predicting mitotic index in vivo to quantitatively predict human cell doubling time in vitro and tumor doubling time in vivo quantitatively, which allowed us to compare our growth rate predictions to clinically observed tumor doubling times in breast cancer. This is in contrast to previous growth‐related metrics, which are semi‐quantitative.^5^, ^6^, ^7^ It is also important to recognize that our growth rate predictor is a fixed‐parameter regression model, meaning that there are no free parameters or “wiggle room” to use for fitting data. Thus, the agreement between our predicted growth rates and clinically measured growth rates across breast cancer subtypes was not fitted but comes entirely from the theoretical derivation of our model.

In summary, the work presented in this study takes the first steps toward identifying the molecular events underlying the natural history of primary breast cancer and presents a model to estimate breast cancer growth rate completely from RNA‐seq data. The methods presented in this work are general and can be used to investigate progression and growth across a variety of cancer types. In breast cancer, although the combination of progression score and growth rate were largely uninformative for patient survival, this may not be the case for cancers that are considered to be “proliferation informative,” such as Kidney Renal Clear Cell Carcinoma or Adenoid cystic carcinoma (ACC).^23^

## CONFLICT OF INTEREST

The authors declare no conflicts of interest.

## AUTHOR CONTRIBUTIONS

Daniel Cook designed the study, performed the analysis, and wrote the paper. Jonatan Kallus and Rebecka Jörnsten performed the survival analysis and gave statistical consultation. Jens Nielsen supervised the work. Daniel Cook, Jonatan Kallus, Rebecka Jörnsten, and Jens Nielsen edited the paper.

## ETHICS APPROVAL

No ethics approval is required because this study does not generate new data or present patient‐identifiable information.

## Supporting information

Tables S1‐S5‐Figs S1‐S4‐Data S1

## Data Availability

The results shown here are in part based upon data generated by the TCGA Research Network: http://cancergenome.nih.gov/ using the breast cancer transcriptomic data set (BRCA). Additional publicly available RNA‐seq datasets used in this study include the SCANB transcriptomic dataset (available at https://www.ncbi.nlm.nih.gov/geo/ with GEO accession: http://www.ncbi.nlm.nih.gov/geo/query/acc.cgi?acc=GSE96058) and the NCI60 cell line panel (available online at https://data.broadinstitute.org/ccle/).
