# Supplementary material for: Molecular natural history of breast cancer: Leveraging transcriptomics to predict breast cancer progression and aggressiveness
Source: Cancer Med. 2020 Mar 23;9(10):3551–62. doi: 10.1002/cam4.2996 (PMC7221450; doi:10.1002/cam4.2996)
Supplement: Supplementary file 1 — Tables S1‐S5‐Figs S1‐S4‐Data S1 [file CAM4-9-3551-s001.docx]

Supplemental Information for

Molecular natural history of breast cancer: leveraging transcriptomics to predict breast cancer progression and aggressiveness

**Table S1.** Cellular and tumor doubling times for prostate cancers.

| Specimen Number | Tumor Number | Tumor volume (cc) | Maximum cellular growth rate (1/months) | Cellular doubling time (months) | Average BrdU LI % | Predicted total growth rate (1/months) | Predicted tumor doubling time (months) | PSA doubling time (months) |
| --- | --- | --- | --- | --- | --- | --- | --- | --- |
| PBr4 | T1 | 2.823 | 0.542 | 1.28 | 5.67 | 0.030 | 23.3 | 18.4 |
|  | T2 | 0.121 | 0.213 | 3.26 | 3.40 |  |  |  |
| PBr6 | T1 | 0.656 | 0.408 | 1.70 | 3.15 | 0.013 | 54.0 | 22.8 |
| PBr18 | T1 | 0.015 | 0.568 | 1.22 | 5.45 | 0.026 | 26.9 | 27.8 |
|  | T2 | 0.842 | 0.286 | 2.42 | 3.75 |  |  |  |
|  | T3 | 0.755 | 0.459 | 1.51 | 9.25 |  |  |  |
| PBr16 | T1 | 7.642 | 0.525 | 1.32 | 4.90 | 0.026 | 26.9 | 32.0 |

**Table S2.** Predicted and experimental tumor volume doubling times across cancer types.

| Cancer Type | Predicted Median Doubling Time (days) | Observed  Doubling Time  Median (Range) |
| --- | --- | --- |
| LIHC | 107 | 86 (11-851) |
| PRAD | 293 | 306 (83-1086) |
| PAAD | 48 | 144 (20-1350) |
| BRCA | 29 | 141 (41-825) |
| OV | 10 | (50-150) |
| GBM | 9 | 30 (1-8021) |

**Table S3.** Coefficients relating the log-transformed expression level of these 23 genes with log-transformed growth rate.

| **Ensembl_ID** | **HGNC_symbol** | **Regression_coeff** |
| --- | --- | --- |
| Intercept | Intercept | 8.519441058 |
| ENSG00000162692 | VCAM1 | -0.018046163 |
| ENSG00000162734 | PEA15 | 0.617519055 |
| ENSG00000198892 | SHISA4 | -0.182070263 |
| ENSG00000143862 | ARL8A | 0.01001093 |
| ENSG00000170448 | NFXL1 | 0.203040747 |
| ENSG00000128050 | PAICS | 0.186982757 |
| ENSG00000243199 | ENSG00000243199 | 1.299116077 |
| ENSG00000256043 | CTSO | -0.056710589 |
| ENSG00000070614 | NDST1 | 0.101654892 |
| ENSG00000145949 | MYLK4 | 0.317962014 |
| ENSG00000215374 | FAM66B | 0.375734686 |
| ENSG00000180155 | LYNX1 | 0.034718798 |
| ENSG00000149054 | ZNF215 | 0.049124221 |
| ENSG00000166788 | SAAL1 | -0.763487559 |
| ENSG00000110446 | SLC15A3 | -0.027566713 |
| ENSG00000198056 | PRIM1 | -0.581945895 |
| ENSG00000111011 | RSRC2 | 0.161798545 |
| ENSG00000132341 | RAN | -1.415227108 |
| ENSG00000177192 | PUS1 | -0.543590512 |
| ENSG00000104064 | GABPB1 | -0.079692462 |
| ENSG00000263350 | ENSG00000263350 | 0.652673562 |
| ENSG00000269420 | PLA2G4C-AS1 | 0.188810736 |
| ENSG00000167747 | C19orf48 | -0.353881187 |

**Table S4.** Genes used to construct the PCNA metagene. Median expression of all genes was used as the expression of the PCNA metagene.

| **Hgnc_symbol** | **Ensembl_gene_id** |
| --- | --- |
| PCNA | ENSG00000132646 |
| CKS2 | ENSG00000123975 |
| NUSAP1 | ENSG00000137804 |
| RRM2 | ENSG00000171848 |
| ZWINT | ENSG00000122952 |
| PRC1 | ENSG00000198901 |
| TFDP1 | ENSG00000198176 |
| CCNA2 | ENSG00000145386 |
| CCNB1 | ENSG00000134057 |
| MELK | ENSG00000165304 |
| TPX2 | ENSG00000088325 |
| BIRC5 | ENSG00000089685 |
| NCAPG2 | ENSG00000146918 |
| RFWD3 | ENSG00000168411 |
| TACC3 | ENSG00000013810 |
| CDC2 | ENSG00000170312 |
| KIAA0101 | ENSG00000166803 |
| MCM2 | ENSG00000073111 |
| MCM5 | ENSG00000100297 |
| ASF1B | ENSG00000105011 |
| CCNB2 | ENSG00000157456 |
| GTPBP2 | ENSG00000172432 |
| KIF20A | ENSG00000112984 |
| PTTG1 | ENSG00000164611 |
| AURKA | ENSG00000087586 |
| CDC20 | ENSG00000117399 |
| DKFZp762E1312 | ENSG00000123485 |
| FOXM1 | ENSG00000111206 |
| GINS2 | ENSG00000131153 |
| MAD2L1 | ENSG00000164109 |
| UBE2C | ENSG00000175063 |
| GINS1 | ENSG00000101003 |
| MCM6 | ENSG00000076003 |
| NCAPD2 | ENSG00000010292 |
| NUP37 | ENSG00000075188 |
| CKS1B | ENSG00000173207 |
| LOC146909 | NA |
| MCM7 | ENSG00000166508 |
| ARID3A | ENSG00000116017 |
| AURKB | ENSG00000178999 |
| CDCA8 | ENSG00000134690 |
| SNRPD1 | ENSG00000167088 |
| TROAP | ENSG00000135451 |
| C21orf45 | ENSG00000159055 |
| DDX39 | ENSG00000123136 |
| ERAF | ENSG00000169877 |
| ESPL1 | ENSG00000135476 |
| HMBS | ENSG00000256269 |
| LSM6 | ENSG00000164167 |
| MCM4 | ENSG00000104738 |
| NCAPD3 | ENSG00000151503 |
| PPIH | ENSG00000171960 |
| CDC45L | ENSG00000093009 |
| DTL | ENSG00000143476 |
| EPB42 | ENSG00000166947 |
| HMGN2 | ENSG00000198830 |
| MCM3 | ENSG00000112118 |
| PF4 | ENSG00000163737 |
| PPBP | ENSG00000163736 |
| RFC4 | ENSG00000163918 |
| TOP2A | ENSG00000131747 |
| BUB1B | ENSG00000156970 |
| CDT1 | ENSG00000167513 |
| FEN1 | ENSG00000168496 |
| GATA1 | ENSG00000102145 |
| GYPB | ENSG00000250361 |
| HMGB2 | ENSG00000164104 |
| KIF22 | ENSG00000079616 |
| KLF1 | ENSG00000105610 |
| MLF1IP | ENSG00000151725 |
| RFC3 | ENSG00000133119 |
| RHCE | ENSG00000188672 |
| TAL1 | ENSG00000162367 |
| TCF3 | ENSG00000071564 |
| ALAS2 | ENSG00000158578 |
| BZRPL1 | ENSG00000112212 |
| CDCA3 | ENSG00000111665 |
| CENPA | ENSG00000115163 |
| CKLF | ENSG00000217555 |
| GTSE1 | ENSG00000075218 |
| NFE2 | ENSG00000123405 |
| OIP5 | ENSG00000104147 |
| SHCBP1 | ENSG00000171241 |
| SNF8 | ENSG00000159210 |
| SNRPB | ENSG00000125835 |
| SPTA1 | ENSG00000163554 |
| KEL | ENSG00000276615 |
| KIF2C | ENSG00000142945 |
| LYL1 | ENSG00000104903 |
| PSMD9 | ENSG00000110801 |
| RACGAP1 | ENSG00000161800 |
| RPIA | ENSG00000153574 |
| TIMELESS | ENSG00000111602 |
| TRMT5 | ENSG00000126814 |
| TYMS | ENSG00000176890 |
| VRK1 | ENSG00000100749 |
| FBXO7 | ENSG00000100225 |
| H3F3A | ENSG00000163041 |
| NUDT1 | ENSG00000106268 |
| PLEK | ENSG00000115956 |
| POLE2 | ENSG00000100479 |
| RHAG | ENSG00000112077 |
| WHSC1 | ENSG00000109685 |
| APOBEC3B | ENSG00000179750 |
| BPGM | ENSG00000172331 |
| CHAF1A | ENSG00000167670 |
| DNAJC9 | ENSG00000213551 |
| FBXO5 | ENSG00000112029 |
| KIF4A | ENSG00000090889 |
| KLF15 | ENSG00000163884 |
| MKI67 | ENSG00000148773 |
| PGD | ENSG00000142657 |
| RPA3 | ENSG00000106399 |
| SFRS2 | ENSG00000161547 |
| TRIM58 | ENSG00000162722 |
| ADAMTS13 | ENSG00000160323 |
| CDCA4 | ENSG00000170779 |
| CDKN3 | ENSG00000100526 |
| FECH | ENSG00000066926 |
| LBR | ENSG00000143815 |
| LIG1 | ENSG00000105486 |
| LMNB1 | ENSG00000113368 |
| MICB | ENSG00000204516 |
| NUP210 | ENSG00000132182 |
| ORC6L | ENSG00000091651 |
| RAD51AP1 | ENSG00000111247 |
| RHD | ENSG00000187010 |
| SMC4 | ENSG00000113810 |
| GYPA | ENSG00000170180 |
| RPP30 | ENSG00000148688 |
| TRIM10 | ENSG00000235025 |

**Table S5.** Coefficients to associate Ki67-based mitotic index with log-transformed PCNA metagene expression

| **Coefficient** | **Value** |
| --- | --- |
| Intercept | -7.99853 |
| PcnaMetagene | 2.434629 |


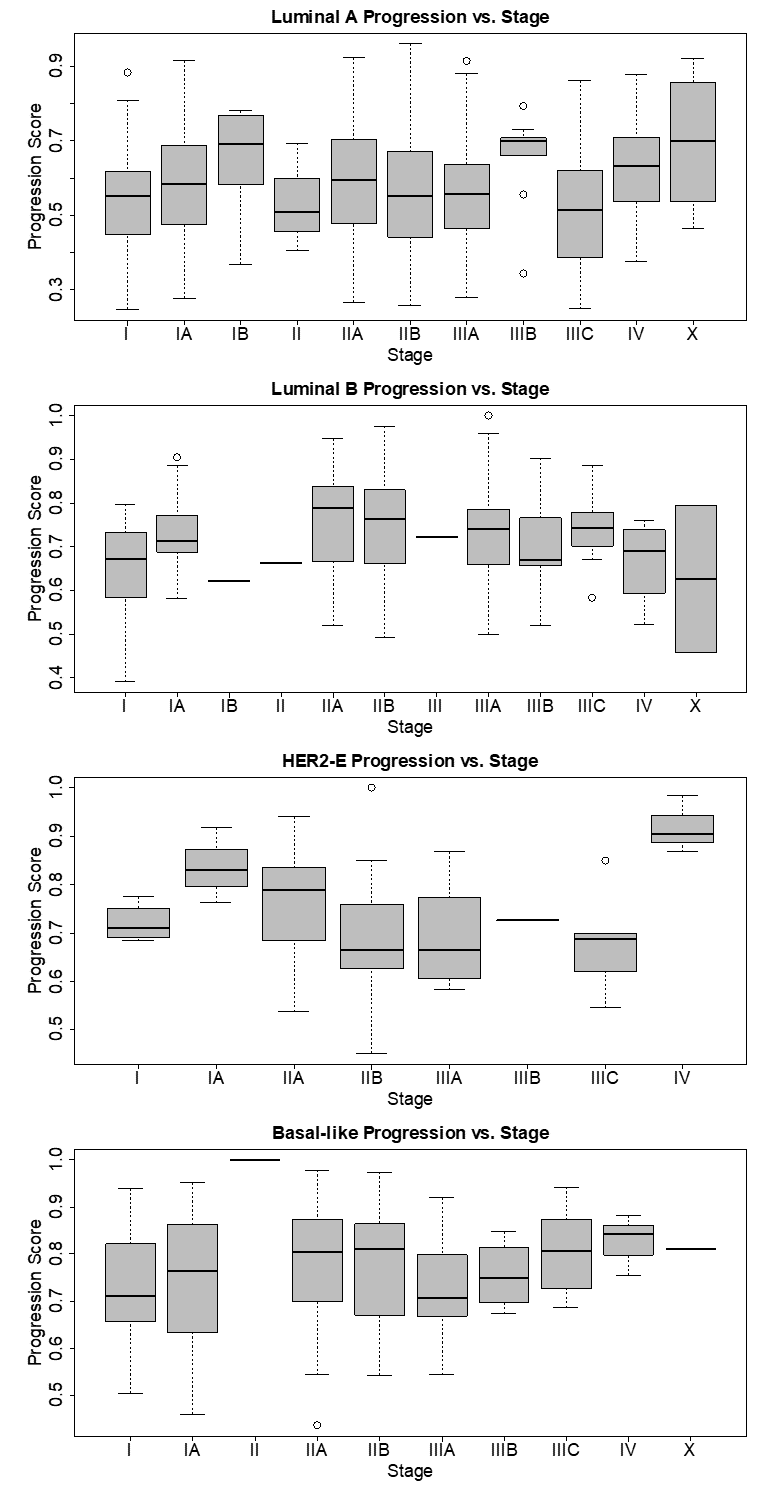


Figure S1. Cancer progression score vs. stage across breast cancer clinical subtypes.


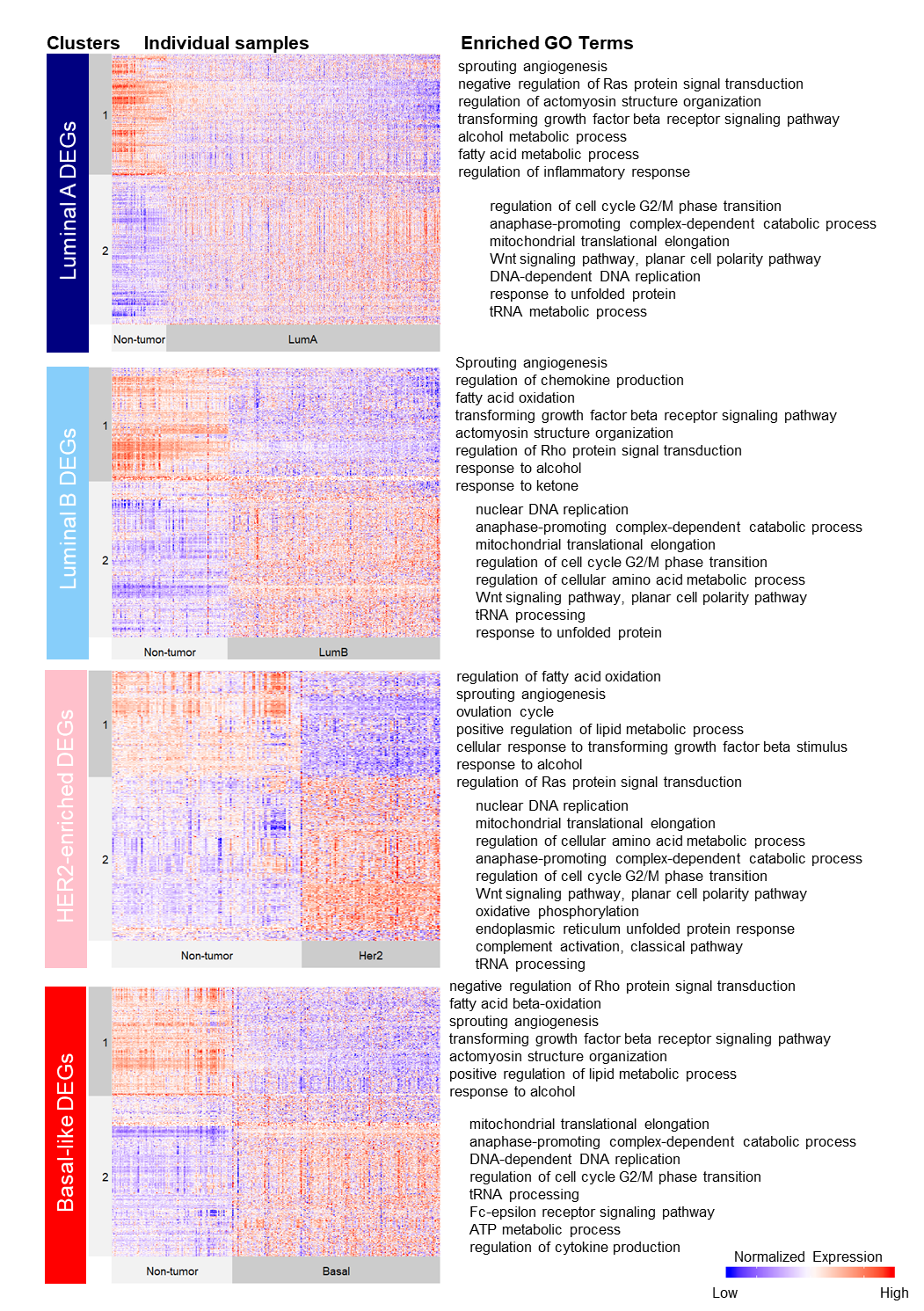


Figure S2: GO term overrepresentation analysis of differentially expressed genes between tumor and stroma for each cancer progression trajectory using PANTHER (25). Overrepresented GO terms were found using metabolic genes measured in the TCGA BRCA data and included in the generic human metabolic model HMR2.0 (26) as a reference background and “GO biological process complete” as the annotation data set. Terms written in grey were found using all human genes as the reference background and “GO biological process complete” as the annotation data set.


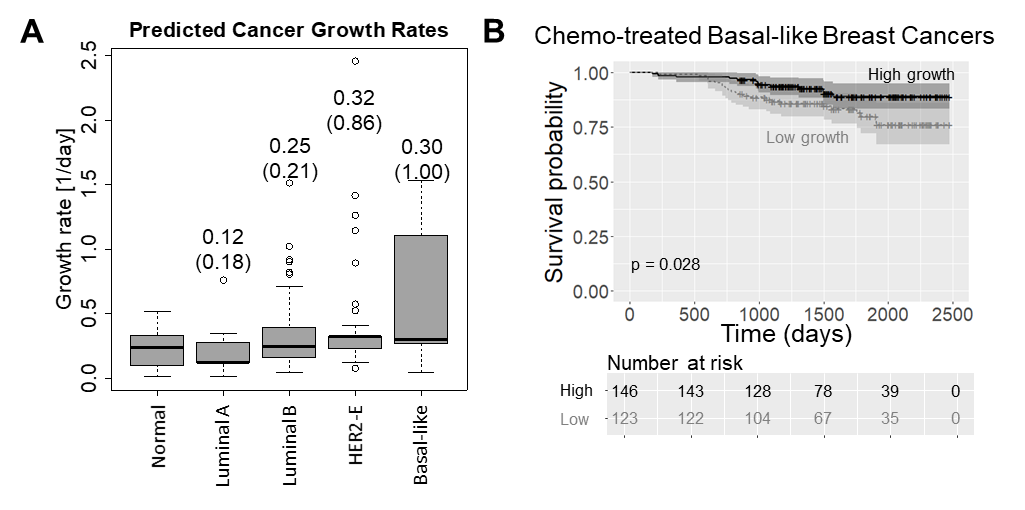


Figure S3. SCAN-B cancer samples. (A) Predicted cancer growth rates for the ~3,700 samples included in the SCAN-B initiative compared to growth rates reported in literature across subtypes. Median growth rate values are shown above the boxplots. Parenthesis give literature growth rate values. (B) KM-plot showing a potential association between growth rate and survival in chemotherapy-treated patients with Basal-like breast cancer. Chemotherapy-treated patients with Basal-like tumors were classified as “high growth” (above median growth rate of this subset of tumors) or “low growth” (below median growth rate of this subset of tumors). A log-rank test was used to calculate a p-value to statistically compare survival curves.

Data file S1. Genes differentially expressed during progression in luminal, HER2-enriched, and basal-like breast cancers. This Excel workbook contains lists of differentially expressed genes and their mean fold-change during each progression period (CPS = 0.2 – 0.9). This fold-change value is based on TPM values and has not been log-transformed.

Additional Supplemental files. Computer code to replicate results. Includes Predict-Progression-BRCA.R, which calls: Predict-Growth-Rates.R, Predict-Mitotic-Index.R, ki67_pred_lm.txt, nci60_growthRegressionCoeff_updated.txt, and venet_2011_PcnaGenes.txt
